# Efficacy and safety of Bacillus Calmette-Guerin for bladder cancer: A protocol of systematic review

**DOI:** 10.1097/MD.0000000000021930

**Published:** 2020-08-28

**Authors:** Zhi-hui Zhang, Lei Yin, Ling-ling Zhang, Jing Song

**Affiliations:** Department of Urology, The Affiliated Hongqi Hospital of Mudanjiang Medical University, Mudanjiang, China.

**Keywords:** Bacillus Calmette-Guerin, bladder cancer, efficacy, safety

## Abstract

**Background:**

This study will systematically assess the efficacy and safety of Bacillus Calmette-Guerin (BCG) for patients with bladder cancer (BC).

**Methods:**

Literature searches will be performed in multiple electronic databases from inception to present: MEDLINE, EMBASE, CINAHL, Science Direct, Cochrane Library, Web of Science, and China National Knowledge Infrastructure. We will also examine grey literature through identifying conference proceedings, thesis, dissertations, and website of clinical trials registry. Two investigators will independently scan all citation titles, abstracts, and full-text studies. The study quality will be assessed by Cochrane Risk of Bias Tool. If possible, we will perform meta-analysis. Additional analyses will be carried out to test the potential sources of heterogeneity among included trials.

**Results:**

The present study will summarize high quality trials on investigating the efficacy and safety of BCG for patients with BC.

**Conclusion:**

The results of this study will supply helpful evidence to determine whether BCG is effective or not for BC.

**Study registration number:**

INPLASY202070042.

## 1 Introduction

Bladder cancer (BC) is one of the most common and prevalent urological cancers globally.^[1–5]^ It is reported that about 400,000 new cases diagnosed annually, and most of them were diagnosed as BC at an age of 65 to 70 years.^[6,7]^ Its incidence is steadily rising worldwide, with incidence rates of 3 to 4 times more in men than in women.^[6,7]^ The main risk factors include cigarette smoking, age, sex, race, family history, chemicals, chronic bladder problems, arsenic exposure, and cyclophosphamide, or pioglitazone, or schistosomiasis usage.^[8–15]^ Understanding the risk factors for this disorder is paramount to help BC treatment and prevention.

Bacillus Calmette-Guerin (BCG) is widely utilized for the management of patients with BC.^[16–18]^ Although a variety of clinical trials reported that BCG could treat BC,^[19–23]^ there is still little literature evidence to specifically and systematically support BCG for BC. Therefore, this systematic review aims to particularly investigate the efficacy and safety of BCG for BC.

## 2 Methods

### 2.1 Study registration

This study was registered on INPLASY202070042. We have reported the present study protocol according to the guideline of Preferred Reporting Items for Systematic Reviews and Meta-Analysis Protocol statement.^[24]^

### 2.2 Eligibility criteria for included studies

This study only includes randomized controlled trials (RCTs) of BCG for patients with BC, regardless language and publication time.

This study will include participants who were diagnosed as BC, in spite of their educational background, economic status, and stages of BC.

Patients who were treated with BCG for BC will be included. Patients who received other treatments will be selected as a comparator, except BCG.

Outcomes consist of pathological complete response, overall survival, progression-free survival, time to progression, recurrence-free survival, disease-free survival, and adverse events.

### 2.3 Strategy of literature searches

The primary source of literatures will be searched from inception to present in MEDLINE, EMBASE, CINAHL, Science Direct, Cochrane Library, Web of Science, and China National Knowledge Infrastructure. The secondary source of potential records will be identified from grey literatures, such as conference proceedings, thesis/dissertations, and clinical trials registry. We build a preliminary search strategy of Cochrane Library (Table 1). We will adapt similar search strategy for other electronic databases to avoid missing potential studies.

**Table 1:**
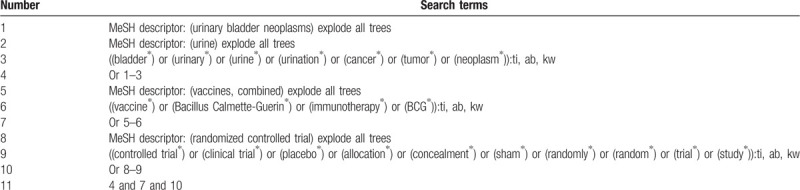
Search strategy of Cochrane Library.

### 2.4 Study selection

All searched records will be imported to EndNote X7, and all duplicates will be removed. Two authors will examine titles/abstracts of all citations to exclude unrelated studies. Full papers of remaining studies will be further identified and evaluated according to all inclusion criteria. Any different views will be solved with the help of another author through discussion. A detailed process of study selection is presented in a flow diagram.

### 2.5 Data extraction process

For all included studies, data will be extracted using a pilot tested data extraction form. It includes primary author, time of publication, trial setting, trial methods, country, trial population, age, eligibility criteria, treatments, controls, comodalities, study limitations, study quality, outcomes, study findings, and other important data. Two authors will independently extract data from each eligible trial, and all divisions will be solved by a third author through discussion.

### 2.6 Dealing with missing data

Whenever insufficient or missing data are found, we will contact primary trial authors to obtain it. If we cannot receive reply, only available data will be analyzed using an intention-to-treat analysis.

### 2.7 Study quality assessment

Study quality of all included trials will be appraised using Cochrane risk of bias tool. This tool will evaluate 7 domains, and each one is rated as low, unclear, or high risk of bias. We will clear up any confusion with the help of a third author through discussion.

### 2.8 Statistical analysis

This study will employ RevMan 5.3 software to perform statistical analysis. All continuous outcome indicators will be expressed as weighted mean difference or standardized mean difference and 95% confidence intervals (CIs). All dichotomous outcome indicators will be showed as risk ratio and 95% CIs. We will quantify statistical heterogeneity using *I*^2^ test. If *I*^2^ ≤ 50%, we will pool outcome data using a fixed-effects model, and we will carry out meta-analysis if sufficient data on the same outcome is extracted. If *I*^2^ > 50%, we will synthesize outcome data using a random-effects model. In addition, we will perform a subgroup analysis to examine its possible heterogeneity sources.

A subgroup analysis will be undertaken based on the differences in types of treatments, comparators, and study quality. A sensitivity analysis will be carried out to investigate the stability of study findings by taking away trials with low quality. If data permits, reporting bias will be evaluated by inspection of the funnel plot^[25]^ and Egger regression test.^[26]^

### 2.9 Ethics and dissemination

This study will only extract data from published trials, thus, no ethic approval is needed. We will publish this study on a peer-reviewed journal.

## 3 Discussion

BC is a very common urological cancer around the worldwide.^[1–4]^ Studies suggested that BCG has been widely utilized to treat patients with BC.^[16–23]^ However, there is still insufficient evidence to support BCG for BC. Therefore, this study will specifically appraise the efficacy and safety of BCG for the treatment of BC systematically and comprehensively. It will synthesize current available data to provide evidence and inform beneficial information for both patients and clinicians.

## Author contributions

**Conceptualization:** Lei Yin, Jing Song.

**Data curation:** Zhi-hui Zhang, Ling-ling Zhang, Jing Song.

**Formal analysis:** Zhi-hui Zhang, Lei Yin, Ling-ling Zhang, Jing Song.

**Investigation:** Jing Song.

**Methodology:** Zhi-hui Zhang, Ling-ling Zhang.

**Project administration:** Jing Song.

**Resources:** Zhi-hui Zhang, Lei Yin, Ling-ling Zhang.

**Software:** Zhi-hui Zhang, Lei Yin, Ling-ling Zhang.

**Supervision:** Jing Song.

**Validation:** Zhi-hui Zhang, Jing Song.

**Visualization:** Zhi-hui Zhang, Lei Yin, Ling-ling Zhang, Jing Song.

**Writing – original draft:** Zhi-hui Zhang, Lei Yin, Jing Song.

**Writing – review & editing:** Zhi-hui Zhang, Ling-ling Zhang, Jing Song.

## References

[1] Cumberbatch MGK Noon AP Epidemiology, aetiology and screening of bladder cancer. Transl Androl Urol 2019;8:5 11. 30976562 10.21037/tau.2018.09.11 PMC6414346

[2] Schulster M Bladder Cancer Academy 2019 selected summaries. Rev Urol 2019;21:23 8. 31239827 PMC6585182

[3] Saginala K Barsouk A Aluru JS Epidemiology of bladder cancer. Med Sci (Basel) 2020;8:15. 10.3390/medsci8010015 PMC7151633 32183076

[4] Karl A Cancer of the urinary bladder: epidemiology, etiology, diagnosis of non-muscle invasive bladder cancer. MMW Fortschr Med 2018;160:43 6. 10.1007/s15006-018-1234-1 30542861

[5] Bray F Ferlay J Soerjomataram I Global cancer statistics 2018: GLOBOCAN estimates of incidence and mortality worldwide for 36 cancers in 185 countries. CA Cancer J Clin 2018;68:394 424. 30207593 10.3322/caac.21492

[6] Antoni S Ferlay J Soerjomataram I Bladder cancer incidence and mortality: a global overview and recent trends. Eur Urol 2017;71:96 108. 27370177 10.1016/j.eururo.2016.06.010

[7] Sanli O Dobruch J Knowles MA Bladder cancer. Nat Rev Dis Primers 2017;3:17022. 28406148 10.1038/nrdp.2017.22

[8] Burger M Catto JW Dalbagni G Epidemiology and risk factors of urothelial bladder cancer. Eur Urol 2013;63:234 41. 22877502 10.1016/j.eururo.2012.07.033

[9] Radosavljević V Ilić M Janković S Epidemiology and risk factors for the onset of urinary bladder cancer. Vojnosanit Pregl 2003;60:195 201. 12852163 10.2298/vsp0302195r

[10] Zaghloul MS Bladder cancer and schistosomiasis. J Egypt Natl Canc Inst 2012;24:151 9. 23159285 10.1016/j.jnci.2012.08.002

[11] Mehtälä J Khanfir H Bennett D Pioglitazone use and risk of bladder cancer: a systematic literature review and meta-analysis of observational studies. Diabetol Int 2018;10:24 36. 30800561 10.1007/s13340-018-0360-4 PMC6357234

[12] Knight A Askling J Granath F Urinary bladder cancer in Wegener's granulomatosis: risks and relation to cyclophosphamide. Ann Rheum Dis 2004;63:1307 11. 15130900 10.1136/ard.2003.019125 PMC1754772

[13] Fernández MI Valdebenito P Delgado I Impact of arsenic exposure on clinicopathological characteristics of bladder cancer: a comparative study between patients from an arsenic-exposed region and nonexposed reference sites. Urol Oncol 2020;38:40.e1 7. 10.1016/j.urolonc.2019.09.013 31630994

[14] Steinmaus C Ferreccio C Acevedo J Increased lung and bladder cancer incidence in adults after in utero and early-life arsenic exposure. Cancer Epidemiol Biomarkers Prev 2014;23:1529 38. 24859871 10.1158/1055-9965.EPI-14-0059 PMC4344186

[15] Hirao Y Kim WJ Fujimoto K Environmental factors promoting bladder cancer. Curr Opin Urol 2009;19:494 9. 19553820 10.1097/MOU.0b013e32832eb4ef

[16] Guallar-Garrido S Julián E Bacillus Calmette-Guérin (BCG) therapy for bladder cancer: an update. Immunotargets Ther 2020;9:1 1. 32104666 10.2147/ITT.S202006 PMC7025668

[17] Kikuchi E Hayakawa N Fukumoto K Bacillus Calmette-Guérin-unresponsive non-muscle-invasive bladder cancer: its definition and future therapeutic strategies. Int J Urol 2020;27:108 16. 31793703 10.1111/iju.14153

[18] Larsen ES Joensen UN Poulsen AM Bacillus Calmette-Guérin immunotherapy for bladder cancer: a review of immunological aspects, clinical effects and BCG infections. APMIS 2020;128:92 103. 31755155 10.1111/apm.13011

[19] Yokomizo A Kanimoto Y Okamura T Randomized controlled study of the efficacy, safety and quality of life with low dose bacillus Calmette-Guérin instillation therapy for nonmuscle invasive bladder cancer. J Urol 2016;195:41 6. 26307162 10.1016/j.juro.2015.08.075

[20] Duchek M Johansson R Jahnson S Bacillus Calmette-Guérin is superior to a combination of epirubicin and interferon-alpha2b in the intravesical treatment of patients with stage T1 urinary bladder cancer. A prospective, randomized, Nordic study. Eur Urol 2010;57:25 31. 19819617 10.1016/j.eururo.2009.09.038

[21] Rentsch CA Birkhäuser FD Biot C Bacillus Calmette-Guérin strain differences have an impact on clinical outcome in bladder cancer immunotherapy. Eur Urol 2014;66:677 88. 24674149 10.1016/j.eururo.2014.02.061

[22] Porena M Del Zingaro M Lazzeri M Bacillus Calmette-Guérin versus gemcitabine for intravesical therapy in high-risk superficial bladder cancer: a randomised prospective study. Urol Int 2010;84:23 7. 20173364 10.1159/000273461

[23] Colombel M Saint F Chopin D The effect of ofloxacin on bacillus calmette-guerin induced toxicity in patients with superficial bladder cancer: results of a randomized, prospective, double-blind, placebo controlled, multicenter study. J Urol 2006;176:935 9. 16890660 10.1016/j.juro.2006.04.104

[24] Shamseer L Moher D Clarke M PRISMA-P Group. Preferred reporting items for systematic review and meta-analysis protocols (PRISMA-P) 2015: elaboration and explanation. BMJ 2015;349:g7647. 10.1136/bmj.g7647 25555855

[25] Sutton AJ Duval SJ Tweedie RL Empirical assessment of effect of publication bias on meta-analyses. BMJ 2000;320:1574 7. 10845965 10.1136/bmj.320.7249.1574 PMC27401

[26] Egger M Davey Smith G Schneider M Bias in meta-analysis detected by a simple, graphical test. BMJ 1997;315:629 34. 9310563 10.1136/bmj.315.7109.629 PMC2127453

